# An Exploration of e-Cigarette–Related Search Items on YouTube: Network Analysis

**DOI:** 10.2196/30679

**Published:** 2022-01-27

**Authors:** Hassan Dashtian, Dhiraj Murthy, Grace Kong

**Affiliations:** 1 The Computational Media Lab and School of Journalism and Media The University of Texas at Austin Austin, TX United States; 2 The Department of Psychiatry at Yale School of Medicine New Haven, CT United States

**Keywords:** electronic nicotine delivery systems, vaping, social media, search engine, natural language processing, social network analysis

## Abstract

**Background:**

e-Cigarette use among youth is high, which may be due in part to pro–e-cigarette content on social media such as YouTube. YouTube is also a valuable resource for learning about e-cigarette use, trends, marketing, and e-cigarette user perceptions. However, there is a lack of understanding on how similar e-cigarette–related search items result in similar or relatively mutually exclusive search results. This study uses novel methods to evaluate the relationship between e-cigarette–related search items and results.

**Objective:**

The aim of this study is to apply network modeling and rule-based classification to characterize the relationships between e-cigarette–related search items on YouTube and gauge the level of importance of each search item as part of an e-cigarette information network on YouTube.

**Methods:**

We used 16 fictitious YouTube profiles to retrieve 4201 distinct videos from 18 keywords related to e-cigarettes. We used network modeling to represent the relationships between the search items. Moreover, we developed a rule-based classification approach to classify videos. We used betweenness centrality (BC) and correlations between nodes (ie, search items) to help us gain knowledge of the underlying structure of the information network.

**Results:**

By modeling search items and videos as a network, we observed that broad search items such as *e-cig* had the most connections to other search items, and specific search items such as *cigalike* had the least connections. Search items with similar words (eg, *vape* and *vaping*) and search items with similar meaning (eg, *e-liquid* and *e-juice*) yielded a high degree of connectedness. We also found that each node had 18 (SD 34.8) connections (common videos) on average. BC indicated that general search items such as *electronic cigarette* and *vaping* had high importance in the network (BC=0.00836). Our rule-based classification sorted videos into four categories: e-cigarette devices (34%-57%), cannabis vaping (16%-28%), e-liquid (14%-37%), and *other* (8%-22%).

**Conclusions:**

Our findings indicate that search items on YouTube have unique relationships that vary in strength and importance. Our methods can not only be used to successfully identify the important, overlapping, and unique e-cigarette–related search items but also help determine which search items are more likely to act as a gateway to e-cigarette–related content.

## Introduction

### Background

e-Cigarette use has grown exponentially since its introduction to the US market in 2007. Currently, e-cigarettes are the second most used tobacco product (4.5%), following cigarettes (14%), among adults [1]. The highest use is among young adults aged 18 to 24 years (9.3%) [1]. A concerning trend is that US adolescents have been using e-cigarettes at an alarmingly high rate; 19.6% of high school students reported using e-cigarettes in the past month (the cigarette smoking rate is 4.6%), and 38.9% of the past-month users were using e-cigarettes for ≥20 days [2]. Similar trends in e-cigarette use have been reported in other countries. For example, e-cigarette use ranges from 0.2% to 27% in European countries [3]. A survey conducted by Wang et al [4] found that a quarter of young Chinese adults had used e-cigarettes. Given the high rates of e-cigarette use worldwide, particularly among young people, it is imperative to understand the appeal, use patterns, and marketing associated with the use of e-cigarettes to inform prevention strategies.

### YouTube and e-Cigarettes

Analysis of social media platforms such as YouTube, which are widely used by youth [5] and have also been shown to have high e-cigarette content [6], could provide insights into e-cigarette use trends and marketing strategies that youth are exposed to. YouTube is a free web-based video streaming service that allows users to view and upload videos, post comments, and rate videos. Users can also interact by subscribing to each other’s YouTube channels and sharing opinions on the video content through writing comments on videos and *liking* or *disliking* videos. YouTube has 2 billion users, which is a third of all internet users, and people currently spend >1 billion hours per day watching web-based videos on the platform [7]. YouTube, along with Facebook, continues to dominate web-based social media use in 2021. Although social media platforms, most notably Instagram, Snapchat, and TikTok, have been very popular among youth, they also actively use YouTube [5].

A concerning finding is that pro–e-cigarette content is readily available on YouTube [6]. An examination of e-cigarette–related YouTube videos from 2012 to 2013 identified 28,000 videos, and these videos were viewed >100 million times, indicating a high level of content consumption [6]. Prior research has also identified that 85% of the pro–e-cigarette content on YouTube is by the e-cigarette industry [8], and the marketing content includes comparisons with cigarettes and emphasizes themes that e-cigarettes are cleaner and cheaper than combustible cigarettes and that they can be used anywhere [9]. Other content areas on YouTube include characterizations of a variety of use behaviors such as vape tricks, which involve using e-cigarettes to blow large, thick amounts of exhaled aerosol (ie, clouds) or shapes [10], and unorthodox use of e-cigarettes, which is manipulating the product to be used for unintended purposes [11,12]. YouTube videos have also been used to understand the way users puff e-cigarettes [13], product characteristics through product reviews [14], the presence of nicotine warning labels [15], and health information regarding e-cigarettes [16], as well as identify and characterize e-cigarette users [10].

### Search Algorithms on YouTube

As YouTube is being used widely to understand e-cigarette–related behaviors, it is important to advance the methods used to obtain and analyze videos. Researchers have searched YouTube for keywords of interest to obtain information on e-cigarettes. Most existing studies that examine e-cigarettes on YouTube have used a range of general search items, such as *electronic cigarette*, *e-cigarette*, *e-cig*, *vaping*, *vape*, *e-liquid*, and *e-juice* [8-10,14,15]. Less known is how well these search items yield videos relevant to e-cigarettes and whether these search items yield comparable or unique results. Currently, there is no guidance for researchers to evaluate how related search items function to yield relevant and parsimonious results on YouTube. Such evaluation is necessary as search items drive the content being shown, which can ultimately shape conclusions reached to inform policy regarding e-cigarettes. Thus, a goal of our study is to use novel methods (eg, network modeling and rule-based classification) to evaluate the association between similar e-cigarette–related search items and search results, as well as to identify which search items act as gateways to e-cigarette–related content.

Both the search and recommendation algorithms on YouTube were structured from the platform’s infancy to drive views of videos [17]. YouTube recommends a series of related videos to users in response to the video currently being played by the users or to a specific search item being used in the search engine. YouTube’s search and recommendation algorithms use machine-learned approaches and are predominantly based on the users’ cumulative viewing experiences. As YouTube’s algorithm is proprietary, the first step in the characterization of e-cigarette–related information on YouTube is to reconstruct the resulting associations between the content of the videos related to this topic that are retrieved from querying YouTube’s search engine. We applied network modeling to study the *relatedness* of search items and the associations between the content of the videos retrieved from different search items. This approach has been successfully used in other studies. Abul-Fottouh et al [18] modeled vaccine-related videos on YouTube as a network to evaluate how the platform recommends vaccination-related videos to its users. Murthy [19] used features of YouTube videos to illustrate and visualize variations in video recommendations based on the language of a video. However, in this study, we used network modeling instead of YouTube’s recommendation system to model YouTube search engine results. Network modeling will help researchers understand how different e-cigarette search items are related in YouTube search results.

We have also used the features of the networks, particularly betweenness centrality (BC) [20], which measures the importance of entities in a network, which have a positional advantage in that they connect the shortest (geodesic) paths between other pairs of entities. A practical application of BC is to determine what search items in the network of information of e-cigarette–related videos are more central and thus provide information seekers with more relevant information in the YouTube search engine. We used correlations between nodes (ie, search items) to characterize the network to identify the relationships between e-cigarette–related search items and the level of importance of each search item in the information network of these search items. Then, we used a rule-based classification to assess whether our search items correctly identified videos related to e-cigarettes.

In summary, the aim of this study is to use network modeling and rule-based text classification methods to understand how the search for e-cigarettes on YouTube is affected by differences in search items. These methods can be used to identify the overlap or uniqueness of search items related to e-cigarettes on YouTube. Assessing whether the search items are parsimonious and whether redundancies can be reduced could inform future work by assisting others in optimizing their search queries to retrieve and analyze information related to e-cigarettes on YouTube videos.

## Methods

**Figure 1:**
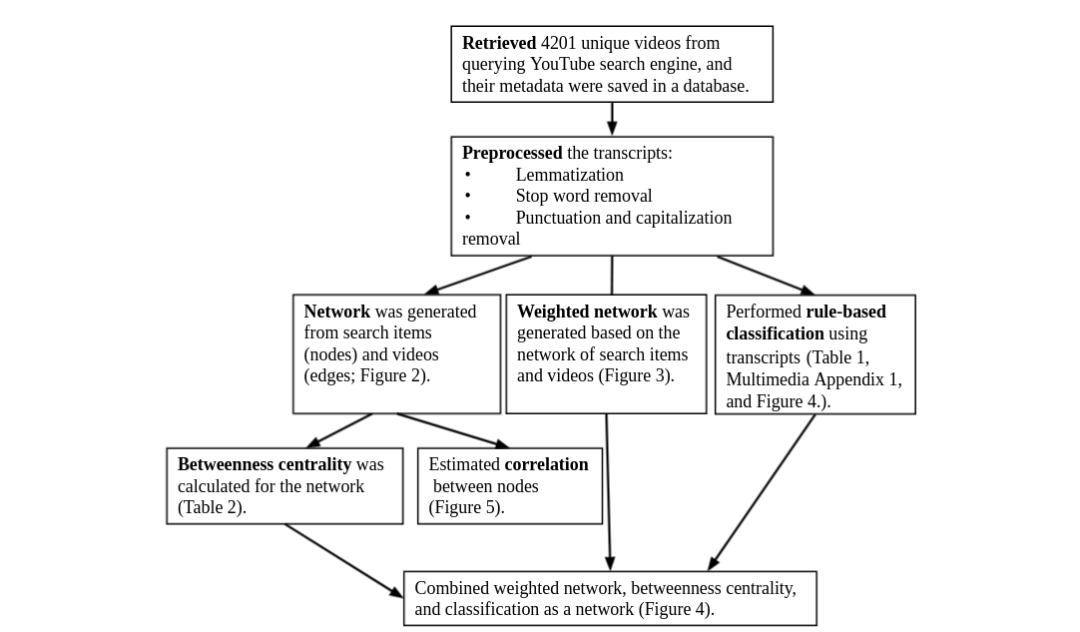
Project architecture; relevant figures and tables are referenced in parentheses.

Figure 1 illustrates the framework we used to collect, process, and analyze YouTube data.

### Video Identification

First, we created fictitious viewer accounts to simulate individuals searching for e-cigarette–related content on YouTube. A separate mobile SIM card was obtained to create a Google account for each fictitious account. The fictitious YouTube profiles comprised African American women and men aged 16 and 24 years (4/16, 25% in total), Hispanic women and men aged 16 and 24 years (4/16, 25% in total), and 2 sets of White women and men aged 16 and 24 years (8/16, 50% in total). We used common African American, Hispanic, and White first and last names to create the fictitious profiles. We created more White profiles to better reflect the e-cigarette use populations [21]. With each of the 16 fictitious viewer profiles, we used a factory-reset Android phone with Orbot (The Tor Project) [22], an app that allows the use of the anonymized Tor IP bridge with any app, to search YouTube for the following keywords related to e-cigarettes in July 2020: *e-cigarette*, *e-cig*, *electronic cigarette*, *e-liquid*, *ENDS*, *e-juice*, *vape*, *vaping*, *vape juice*, *box mods*, *cigalikes*, *disposable e-cigs*, *disposables*, *disposable vape*, *pod mods*, *vape mods*, *vape pens*, and *vape pods*. We factory reset the phone and Tor IP address after collecting data for each user so that any personalization by YouTube was erased. Other researchers have used general search items related to e-cigarettes on YouTube to identify trends in perceptions, use, marketing and sales related to e-cigarettes [8-10,14,15]. On the basis of this prior literature, we chose a single general term related to *e-cigarettes* as our search item to assess whether our methods could be used to evaluate the associations between the results derived from these search items.

Across all search items and for each profile, we requested 140 videos (7 pages with 20 videos on each page) and collected a total of 5875 videos after removing the duplicates across different profiles (see Multimedia Appendix 1 for a breakdown of the collected videos by search item after the duplicates across the profiles were removed). Of those 5875 videos, we further removed 1674 (28.49%) duplicates across different search items, which resulted in our final data set of 4201 (71.51%) unique videos. We did not exclude duplicate videos when generating the network; we merely removed duplicates for classification purposes. For these 4201 videos, we extracted video transcripts directly from the YouTube application programming interface (API). We did not restrict the date of video upload as some videos that were uploaded years ago could still be relevant. The video metadata that we requested from the YouTube API were public; therefore, the Yale Institutional Review Board deemed that institutional review board approval was not required.

### Search Items as a Network

We modeled the resulting videos derived from the search items as a network. We assumed that each search item was a node and each video was an edge. If 2 search items had a connected edge, there was a common video in the resulting video data set. This method helped us to better visualize the connectivity of the search items and visualize the connectivity between search items. We then used social network analysis to study how search queries may be connected to each other. Connections between nodes in a network were a good proxy for the similarity between nodes. A search item’s similarity with others could be measured by comparing the video IDs that we retrieved from querying YouTube.

### BC Analysis

**Figure 2:**
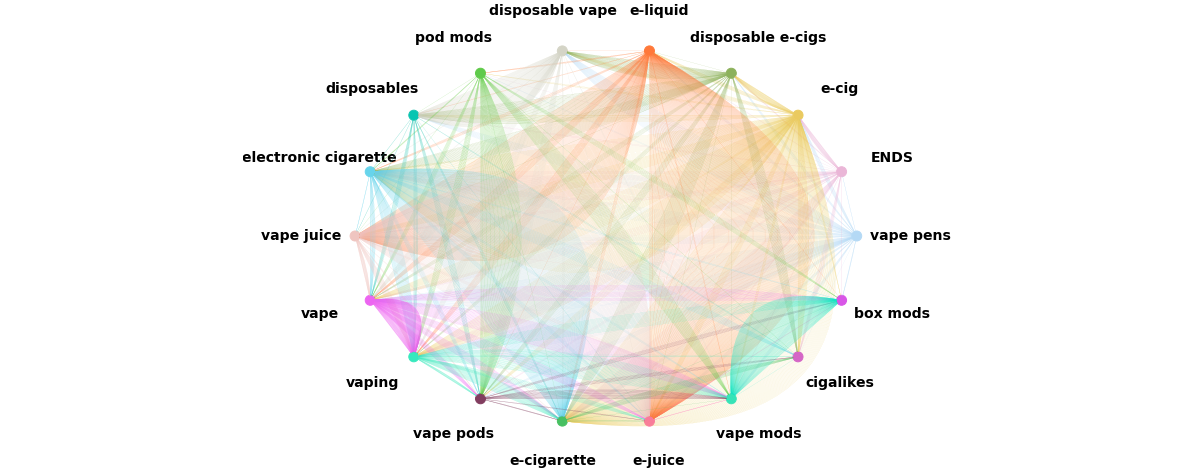
The network of search items and videos from our YouTube searches. Connections (edges) between the nodes (search items) indicate when the same videos resulted from 2 different search items, and a darker shaded edge line indicates a higher frequency of common results. ENDS: electronic nicotine delivery system.

We then identified the nodes that had the most influence on the network of search items using BC, which measures the importance of entities (ie, nodes) in a network, which have a positional advantage in that they connect the shortest (geodesic) paths between other pairs of entities. This metric estimates all the shortest paths between every pair of search items in the network (Figure 2) and then computes the number of times a search item (node) is on the shortest path between 2 other nodes. Nodes with high BC may have considerable influence within a network by virtue of their control over information passing between others. They are also the ones whose removal from the network will most disrupt communications between other nodes as they lie on the largest number of paths connecting nodes.

Ultimately, BC indicates the importance of each node in a network. The BC of a node *v* is the sum—taken over all pairs *(s, t)* of source–target nodes—of the ratio between the number of shortest paths from *s* to *t* passing through *v* and the total number of shortest paths from *s* to *t*. Therefore, nodes with high BC belong to several shortest paths, whereas nodes with low BC belong to few shortest paths. A node with high BC is presumed to be a significant and influential node. An important feature of BC is that it is the measure of the degree to which a node is *between* other nodes in a graph or network, meaning that this node could act as a mediator in the network regardless of the frequency of connectivity [23,24]. This is very important in the context of YouTube as the platform’s recommendation algorithms may use BC as a parameter to recommend videos based on the search items.

### Correlogram

We calculated the correlation coefficient between the nodes (search items) and the edges (number of videos) that linked to each other in the network and visualized the correlation using a correlogram. A correlogram can be used to visualize which nodes behave similarly (positive correlations) or differently (negative correlations).

### Rule-Based Classification

Our methodological approach to classifying the themes of the videos used (1) a custom-developed Python script to extract video transcripts and video metadata, including number of views, title, category on YouTube, number of likes or dislikes, and comments; (2) preprocessing and lemmatization; (3) natural language processing (NLP) methods to extract keywords from video transcripts for their representation while removing stop words from the transcript; and (4) a rule-based classifier to categorize themes.

### Preprocessing Data and Lemmatization

We cleaned the raw data by checking the quality of the raw text. Our preprocessing pipeline involved converting the raw text from the video transcripts into a vectorized form that was easily interpretable by our models. We used the spaCy (Explosion AI) library [25], an open-source NLP software library that comes with pretrained word vectors, to preprocess these transcripts. We used the provided *GloVe* [26] vectors from the *en_core_web_lg* model, which were trained on a variety of internet corpora. The use of word vectors allowed us to provide our models with a meaningful representation in a vectorized form that encoded semantic similarity between words. Ideally, these word vectors would allow our models to use semantic relationships to determine whether the text we were classifying fell within a given theme, such as *e-cigarette device*. We used spaCy’s list of 312 stop words (eg, *and*, *a*, *our*, and *my*) as they did not significantly contribute to the understanding of our data. We also lemmatized the words so that different word conjugations appeared identical to the classifier.

### Rule Set

Approaches for the classification of text included rule-based methods, machine learning–based methods, and hybrid approaches that used both machine learning and rule-based methods [27,28]. We used rule-based methods to classify the text of the transcript of the video to identify whether the search items were indeed identifying e-cigarette videos. Rule-based approaches classify text into classes using a set of custom linguistic rules. These rules direct the algorithm to use semantically relevant elements of a text to identify similar categories based on the content of the texts and documents. Our rule set is detailed in Textbox 1. Indicators for each class were chosen by identifying common words in the video titles, descriptions, and transcripts.

We initially implemented a framework with >4 categories but discovered that some categories had very few cases. Therefore, we grouped categories with low frequencies into *other e-cigarette videos*. This category included videos that did not feature a specific e-cigarette device and were related to health information and news clips that contained news related to e-cigarettes. The other three categories included e-cigarette products featured on videos presented to YouTube users: specific e-cigarette devices that are used for vaping, the liquid that is used in the devices, and cannabis vaping products (Textbox 1). Our rationale for a rule-based method stems from 2 key reasons. First, we use a small number of classes (n=4), and these classes can be easily separated with a rule-based approach, and we do not need to train more sophisticated models to classify the video texts. Related text—specifically video transcripts—usually contain high dimensional nondiscriminative (irrelevant and noisy) words that can result in high computational costs and poor learning performance [29].

**Rule set for theme-based classification of e-cigarette–related videos.**

**Class type and indicators** **(videos can be categorized into >1 category)**

e-Cigarette device

*e-cigarette*, *electronic cigarette*, *e-cig*, *ecig*, *vape pen*, *vapepen*, *vape*, *pod*, *pod mod*, *disposable ecig*, *disposable vape*, *disposable*, *cigalike*, *box mod*, *boxmod*, *juul*, *puff bar*, *cartomizer*, *drip*, *drip tip*, *vape kit*, *mod*, *electronic nicotine delivery system*, *ENDS*, *puffbar*, *puff bar*, *sourin*, *blu*, *smok*, *leaf*, *markten*, *nicotek*, *vuse*, *fin*, *v2*, *21st*, *atomizer*, *RDA*, *RTA*, *cartridge*, *ohm*, *wattage*, *watt*, *drip*, *cartomizer*, *ecig+device*, *vape+device*

e-Liquid

*e-juice*, *e-liquid*, *ejuice*, *eliquid*, *vapejuice*, *vape liquid*, *zamplebox*

Cannabis vaping product**s**

*cheeba*, *dab*, *firefly*, *ganja*, *gpen*, *hemp*, *indica*, *kush*, *marijuana*, *pax*, *pot*, *reefer*, *sativa*, *snoop dogg*, *weed*, *THC*, *cannabis*, *hash*, *wax*, *CBD*

Other e-cigarette videos

These videos included e-cigarette–related videos but did not feature a specific e-cigarette device (eg, news clips and health information videos). We categorized videos that did not fit into any of the classes above into this category.

## Results

### Search Item Network

Figure 2 illustrates the network of 18 search results. In this network, each node (search item) is represented by a unique color, and darker shaded edges indicate a higher frequency of connections. Each line represents a common video (edge) between 2 nodes, indicating that a common video has been found in the search results for both search items. As expected, similar search items had more common videos. For example, *e-cigarette* and *electronic cigarette* had more common edges than *e-juice* and *disposable*.

**Figure 3:**
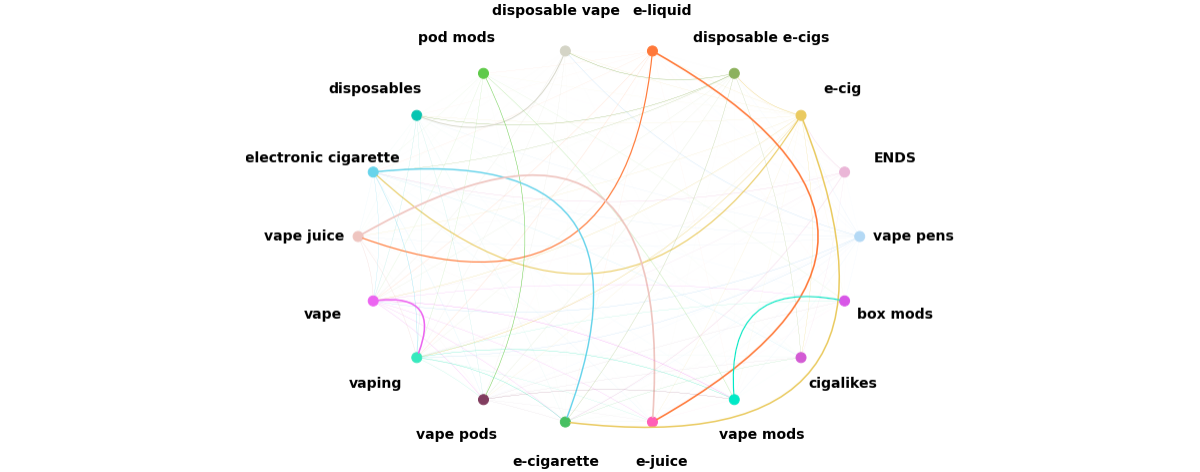
The network of search items and videos from our YouTube searches with weighted edges. The weighted edges, which represent the relative frequency of connections between 2 nodes, are represented by the thickness of each line. ENDS: electronic nicotine delivery system.

Another way of representing the network of videos is to convert the number of connections (ie, edges) to a weight within the network for search items (Figure 3). We assumed that the nodes were connected through weighted edges. We counted the edges (common videos) between 2 nodes (search items) and normalized this number to a weight between 0 and 1, with 1 corresponding to the maximum number of edges between 2 nodes in the network and 0 indicating the minimum number of edges. The thickness of the edges is an indication of the weight, and thicker edges indicate more common videos and a stronger information flow and relationship between the 2 nodes.

From the networks illustrated in Figures 2 and 3, search items with similar words (eg, *vape* and *vaping*) and search items with similar meanings (eg, *e-liquid* and *e-juice*) have strong weighted edges, indicating that YouTube’s search algorithm gave similar search results for these 2 categories. *e-Juice* and *vape juice* had 188 common edges, which was the maximum number of connections between 2 search items. *e-Juice* and *e-liquid* had 179 common edges, which was the second-largest number of connections. *Cigalike* had the lowest overall connections (n=82), whereas *e-cig* had the highest total connections (n=505). Search items *vape pens*, *ENDS*, and *e-cig* had at least one connected edge to other search items. *e-Cig* had the most connected edges to others (mean 29.7, SD 45.2), and *e-cigarette* had the second-highest connected edges (mean 27.6, SD 52.9). *Cigalikes* and *pod mods* had 4.8 and 6.6 common edges with other search items, respectively, which were the lowest connected edges. On average, each node had 18.0 (SD 34.8) connections, indicating that there was a large variation in the number of connections between search items. This large SD could also be because of the fact that some of the search items were not connected (ie, there were no common videos between them). For example, *cigalikes* and *box mods* or *disposable vape* and *pod mods* had no connections. The complete list of the average connections is detailed in Table 1.

**Table 1:** Average number of connections (videos) for each node (search item) in the network and BC^a^ of each node in the network.

Search item	Number of connections, mean (SD)	BC
*e-cig*	29.7 (45.2)	0.00836
*e-cigarette*	27.6 (52.9)	0.00836
*vaping*	26.2 (39.6)	0.00836
*electronic cigarette*	25.4 (45.1)	0.00836
*e-juice*	25.2 (59.7)	0.00232
*vape*	23.9 (39.6)	0.00407
*vape juice*	23.8 (52.3)	0.00407
*e-liquid*	21.5 (50.9)	0.00407
*vape mods*	15.2 (32.5)	0.00726
*disposable vape*	13.3 (24.8)	0.00049
*disposables*	11.5 (24.1)	0.00521
*disposable e-cigs*	11.5 (13.0)	0.00396
*box mods*	11.4 (32.4)	0.00056
*vape pens*	10.5 (9.3)	0.00836
*vape pods*	10.2 (11.5)	0.00836
*ENDS^b^*	7.9 (9.4)	0.00836
*pod mods*	6.6 (12.5)	0.00392
*cigalikes*	4.8 (6.4)	0.00105

^a^BC: betweenness centrality.

^b^ENDS: electronic nicotine delivery system.

### BC and Rule-Based Classification

**Figure 4:**
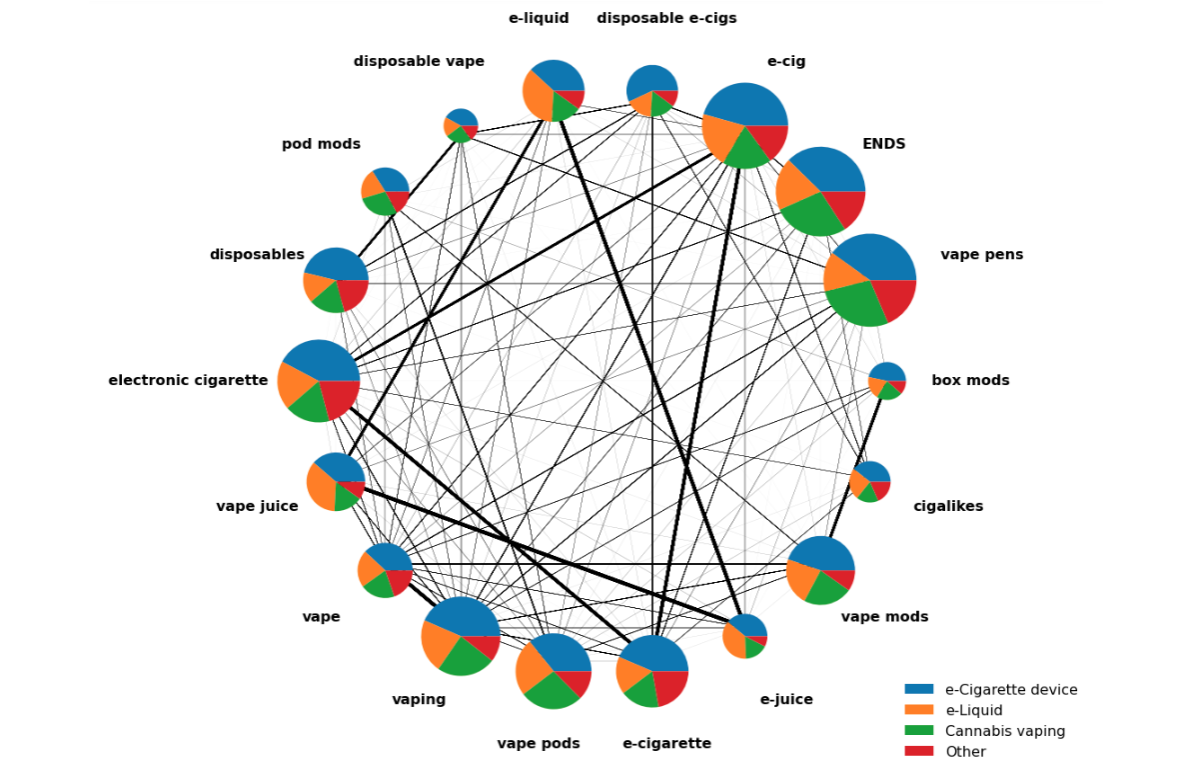
Weighted network of search items. The thickness of an edge (thickness of the lines) indicates the weighted number of common videos between 2 search items. Betweenness centrality (BC) and classes (themes) of videos in search items are also shown. The size of each node (represented as a pie chart) indicates a node’s BC, with a larger size indicating higher BC than a smaller size. Each pie chart shows the percentage of 4 classes in each search item. ENDS: electronic nicotine delivery system.

BC can be used to demonstrate the flow of information and provide a connective fabric between different search items in the network of e-cigarette–related videos. In our study, BC detected the dominant nodes (search items) that occupied a set of intermediate items observed between 2 other search items in the information network constructed with search items and YouTube videos. Generally, nodes with high BC are assumed to have a considerable impact on a network and can be considered *important* nodes as they control the information flow between other nodes. Furthermore, a node with high BC may not have a high connectivity degree. For example, in our network, the *ENDS* node had 7.9 (SD 9.4) connections on average and was considered low relative to other search items; however, its BC was relatively high (Table 1 and Figure 4). High-BC nodes (with a low connectivity degree) can act as bridges between different clusters in a network (ie, subnetworks). Our network-based results indicate that there were subnetworks in the information network of e-cigarette–related videos, with search items such as *ENDS* acting as a bridge between these subnetworks. From a practical perspective, this means that users searching for ENDS could be exposed to ENDS content as well as videos in the *cigalike* and *e-liquid* subnetworks (eg, through YouTube’s recommended videos feature), whereas searches for just *cigalike* or *e-liquid* would be more restricted in terms of both search results and videos being recommended.

In Figure 4, the size of the pie indicates the relative BC values for the network of search items. For instance, a larger pie size indicates greater BC, suggesting that these are important nodes that serve as gateways to most videos in the network of videos derived from our data set. Nodes that did not show high levels of importance are represented by midsize pie charts, and nodes that had a low level of importance are represented by small pie charts. Specifically, the search items *vape pens*, *ENDS*, *e-cig*, *electronic cigarette*, *vaping*, *vape pods*, and *e-cigarette* had the highest BC (Table 1), whereas *disposable vape*, *box mods*, and *cigalikes* had the lowest values and, therefore, were the least important nodes in the network of search items.

Rule-based classification identified that *e-cigarette device* was dominant in all search items (34%-57%), followed by videos on e-liquids (14%-37%), cannabis (16%-28%), and *other* (8%-22%). These classes (labels) are shown in Figure 4. The pie chart, which represents a search item in the network of videos, shows the percentages of videos that belong to 1 of the 4 classes. For example, in the *disposable e-cigs* node, 57% of the videos are labeled as *e-cigarette device*, which is the highest among all the nodes (see Multimedia Appendix 1 for the percentage of classes for each search item). Interestingly, although we did not use search items that were directly related to *cannabis*, all the search items did have a considerable percentage of videos with vaping cannabis content.

**Figure 5:**
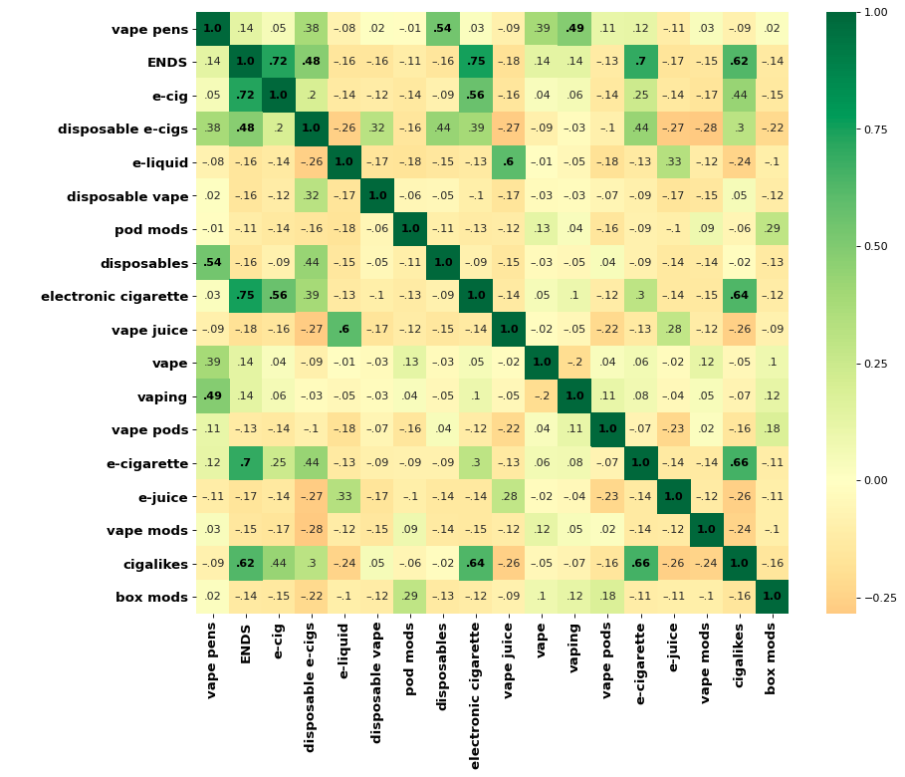
The correlogram of the search item network. Each cell indicates the correlation between the corresponding 2 nodes. The strength of the correlation is represented both by numerical value and by the gradient (represented by background color). More significant correlations with P≤.05 are shown in bold font. ENDS: electronic nicotine delivery system.

Figure 5 shows the correlogram (correlation matrix), with each value indicating the correlation coefficient between the 2 corresponding search items. The magnitude of the correlation is represented by the color and a number on the corresponding square. Most pairs had negative or low positive correlations. The highest positive correlation in the network was observed between *electronic cigarette* and *ENDS* (0.75). The lowest correlation was observed between *vape mods* and *disposable e-cigs* (−0.28), which was the lowest in the network, suggesting that these nodes behave differently.

## Discussion

### Principal Findings

In this study, we used novel methods—such as network analysis and rule-based classification—to evaluate the associations among e-cigarette–related search items on YouTube. We observed that broad search items such as *e-cig* had the most connections to other search items and that specific search items such as *cigalike* had the least connections. Search items with similar words (eg, *vape* and *vaping*) and search items with similar meaning (eg, *e-liquid* and *e-juice*) yielded a high degree of connectedness. We also found that each node (ie, search item) had 18 (SD 34.8) connections (common videos) on average. BC indicated that general search items such as *electronic cigarette* and *vaping* had high importance in the network (0.00836). Our rule-based classification sorted videos into 4 categories: e-cigarette devices, cannabis vaping, e-liquid, and *other*.

Our results show that similar search items that were more specific (eg, *e-liquids* and *e-juice*) derived similar videos and that broad items (eg, *e-cig* and *e-cigarettes*) yielded a wide range of videos that were also identified with other search items. More specific items such as *cigalikes* and *pod mods* were less likely to be connected to other types of videos and identified unique videos that closely represented these items. For example, Massey et al [12] examined specific topics related to e-cigarettes, such as modifications of e-cigarettes, and searched YouTube for 28 phrases related to e-cigarette use as well as items related to modifications such as custom build, modification, and dripping. These research findings suggest that when identifying a specific topic area related to e-cigarettes, both broad items and specific items relevant to the topic area should be used. Our findings also suggest that there is a high level of redundancy in search results between pairs of similar search items such as *ENDS* and *e-cigarettes*, *vape* and *vaping*, and *vape juice* and *e-juice*, which suggests that redundant search items can be removed and the e-cigarette–related YouTube videos retrieved would largely be unaffected.

Our findings suggest that the local connectivity of mutual nodes in the network is important, and we looked at the relationships between a node and its neighbors regardless of its relationship with the corpus. However, we were also interested in finding the search items whose removal from the network would most disrupt the network. We used BC to demonstrate the flow of information between different search items in the network of e-cigarette–related videos to accomplish this. In our information network (Figure 4), search items (nodes) were connected through common videos and, when there was no direct connection—illustrated by a line (edge)—between 2 nodes, a third node might enable connectedness. For instance, a search for *pod mods* on YouTube may return search results of videos that may also show search items such as *vape pods* and *vape mods*. When a user watches a video (retrieved from searching *pod mods*) that occurs in both *pod mods* and *vape pods*, the content of the video can influence the user and help the user consume *vape pods* content. However, as there are no direct common videos between *pod mods* and *disposable vape*, there is no content to guide the user directly toward videos related to *disposable vape* (from retrieved videos from searching *pod mods* only). Thus, a third search item (eg, *vape pods*) is needed for a user to traverse between these 2 nodes. Our network was relatively small but, for large networks, there might be several paths that users can traverse to reach nodes from a starting point in the network of search items. It should be noted that users can access information related to *disposable vape* through a variety of other related search items, such as *disposable*, *vape pens*, and *e-cig* (Figure 3). For the data that we collected, YouTube search results presented no common videos between the *pod mods* and *disposable vape* search items in the 16 fictitious profiles that we created. Ultimately, the search results were time dependent, and our results may not hold true in the future if YouTube’s algorithms or input data change.

In a network, nodes that possess more central positions (higher BC) are more likely to provide informational content with less central members and have a more heterogeneous connection array [29,30]. In our study, search items such as *electronic cigarettes*, *ENDS*, and *vape pens* played this role. Looking at the measure of all search items, there was considerable variation in this value (from 0.00049 to 0.00836). However, with that noted, the overall BC values were low, meaning that connections in the network of search items could be made without intermediaries [31]. Generally, BC highlights the importance of a node in an information network as a transfer point between any pair of nodes. Consider the case in which a YouTube user uses several search items (nodes in our network) to obtain e-cigarette–related content (similar to the framework that we developed in this study). Some search items are more central in the list of search items as they will generate results broadly related to e-cigarettes, whereas more specific, niche items may be needed to access content specific to particular subdomains related to e-cigarettes.

### Limitations and Strengths

The limitations of this study should be noted. Although we examined 18 search items related to e-cigarettes based on the items previously used in other studies [12], we did not use all the potential items used to refer to e-cigarettes to assess what video content users were likely to encounter on YouTube. There are many other items used to refer to e-cigarettes, such as brand-specific names (eg, *Juuling*) that were not used in this study and vernacular that is used to refer to e-cigarette use (eg, *stick*). Future work can use our methods to evaluate other search items to assess whether these search items produce relevant e-cigarette–related videos of interest. Our study findings indicate that search items that have similar meanings or words are likely to have significant overlap in the items of the resulting videos. Therefore, studies would benefit from pilot or exploratory work to determine the best search query items before proceeding with full-scale data collection. It is important to note that actual users search social media platforms for more complex themes rather than single words, such as motivations for using e-cigarettes or concerns about health outcomes related to their use [32]. Future research should extend our methods to assess the search results derived from a combination of words with different themes.

Our rule-based classification of video transcripts provides further information about the network. However, rule-based classification has some limitations as it relies on domain-specific expert knowledge. Therefore, each class may include some features (indicators similar to those in Textbox 1) that have not been incorporated into the selected features of that label. Despite these limitations, this is the first study to examine e-cigarette–related content on YouTube using NLP, video classification, and network modeling.

Another limitation of this study is that we used English-language search items to query the YouTube API. Other languages can be used to search videos on YouTube. Future work should evaluate other search items to assess whether our findings hold true for non–English-language videos.

Our study contributes to the existing literature related to e-cigarettes on YouTube [6,10,12,33,34] and provides tools to explore the search items as an information network. We developed a general framework for acquiring, cleaning, and analyzing the search data related to e-cigarettes on YouTube. Our use of network-based approaches presents a novel approach to the study of e-cigarettes on social media platforms. Our network-based approach highlighted the connections between different search items and enabled us to identify the associations between various e-cigarette–related search items. We used network analytic methods—particularly BC, which provided quantitative measures to better understand e-cigarette–related content on YouTube—to understand the underlying relationships between search items and characterize the information network formed by thousands of videos. Several centrality measures have been developed and are currently being used by search engines and recommendation systems in social media [35]. Although measures such as BC have been used extensively to study health-related content on social media [36], our study is the first to use BC to demonstrate the flow of information between different search items in the network of e-cigarette–related videos.

Importantly, our methods can be used by public health researchers to optimize search results on YouTube to better understand e-cigarette trends. Understanding search items is important as search items drive the information being presented to users. Our methods provide insight into how YouTube’s algorithm selects and presents e-cigarette videos to users.

Surveillance of e-cigarettes on YouTube is crucial to understanding how health information and marketing are being communicated to users and nonusers, particularly among youth. Youth are vulnerable to e-cigarette content on social media [37], and they actively use social media platforms such as YouTube to obtain information, including information on novel uses of e-cigarettes [38]. Thus, having a better understanding of e-cigarette information being shown to youth is an important public health goal, as such information can provide insight into the public health policies needed and the social media platforms that control these algorithms. Given that YouTube’s algorithm is proprietary, we can use these methods to evaluate search results even if YouTube introduces wholly new algorithms to evaluate the connectedness between search items and resulting videos. Our approach can also be applied longitudinally to determine whether YouTube search results change over time and to what extent the network of search items and their properties differ as a function of time. In addition, although we focused on e-cigarette–related content on YouTube in this study, we believe that our approach can be implemented on other platforms. Similarly, Harris et al [39] used network analysis to study the content of tweets and identify Twitter users prominent in the conversation for and against the e-cigarette Twitter campaign of the Chicago Department of Public Health. Future work could also evaluate whether our findings hold true for other social media platforms.

### Conclusions

We found that similar search items (eg, *e-liquids* and *e-juice*) and items with similar word structures (eg, *e-cig* and *e-cigarettes*) yielded similar videos. In addition, general search items such as *e-cig* yielded a broad range of videos that were also identified by other related search items. Ultimately, broader search items act as gateways to the network of e-cigarette–related search items. More specific items such as *cigalikes* and *pod mods* are less likely to be connected to other types of videos and are useful in identifying unique videos that may closely represent more specific, niche e-cigarette–related areas. Our methods can be used as a measure to exclude some search items from future studies, restrict the number of search items, and identify search items that serve as important gateways to broader e-cigarette–related content. Importantly, public health researchers can use our methods to optimize search results on YouTube to better understand how search items related to e-cigarettes drive the content that is shown to youth on this popular social media platform as well as on other social media platforms.

## Appendix

Multimedia Appendix 1 Number of videos retrieved from each search item and the percentage of each class. We retrieved 5875 videos (4201 unique) by searching for 18 search items. There were 1674 duplicate videos between search items.

## Abbreviations

API: application programming interface
BC: betweenness centrality
NLP: natural language processing
