# Supplementary material for: An Exploration of e-Cigarette–Related Search Items on YouTube: Network Analysis
Source: J Med Internet Res. 2022 Jan 27;24(1):e30679. doi: 10.2196/30679 (PMC8832267; doi:10.2196/30679)
Supplement: Multimedia Appendix 1 [file jmir_v24i1e30679_app1.docx]

**Multimedia Appendix**

**Appendix** 1**:** Number of videos retrieved from each search item and percentage of each class. Note: We retrieved 5875 videos (4201 unique) by searching for 18 search items. There are 1674 duplicate videos between search items.

| Search item | Number of videos (n) | e-cigarette (%) | e-liquid  (%) | cannabis  (%) | other  (%) |
| --- | --- | --- | --- | --- | --- |
| vape pens | 338 | 40 | 14 | 27 | 19 |
| ENDS | 317 | 38 | 19 | 28 | 16 |
| e-cig | 359 | 46 | 21 | 18 | 15 |
| disposable e-cigs | 218 | 57 | 17 | 16 | 10 |
| e-liquid | 345 | 38 | 36 | 16 | 10 |
| disposable vape | 323 | 42 | 18 | 25 | 15 |
| pod mods | 264 | 34 | 21 | 28 | 17 |
| disposables | 283 | 46 | 15 | 18 | 21 |
| electronic cigarette | 331 | 42 | 19 | 18 | 21 |
| vape juice | 348 | 39 | 36 | 16 | 10 |
| vape | 373 | 38 | 22 | 21 | 20 |
| vaping | 400 | 43 | 22 | 24 | 11 |
| vape pods | 365 | 36 | 25 | 27 | 13 |
| e-cigarette | 320 | 44 | 17 | 18 | 22 |
| e-juice | 335 | 39 | 37 | 17 | 8 |
| vape mods | 366 | 45 | 22 | 23 | 10 |
| cigalikes | 232 | 39 | 25 | 17 | 18 |
| box mods | 358 | 47 | 19 | 22 | 12 |
